# The Changing Selenium Nutritional Status of Chinese Residents

**DOI:** 10.3390/nu6031103

**Published:** 2014-03-14

**Authors:** Sumei Li, Gary S. Bañuelos, Longhua Wu, Weiming Shi

**Affiliations:** 1 State Key Laboratory of Soil and Sustainable Agriculture, Institute of Soil Science, Chinese Academy of Sciences, Nanjing 210008, China; E-Mails: smli@issas.ac.cn (S.L.); lhwu@issas.ac.cn (L.W.); 2 USDA, Agricultural Research Service, San Joaquin Valley Agricultural Sciences Center, Parlier, CA 93648, USA; E-Mail: Gary.Banuelos@ARS.USDA.GOV

**Keywords:** hair Se content, historical Se data, grain consumption

## Abstract

The selenium (Se) content in human hair is useful as an indicator of human Se intake and status. In this regard, when measuring the hair Se concentrations in Chinese inhabitants across northeast to southeast China, the results indicated that generally 84% of all residents have normal hair Se content. Between the sexes, the average hair Se content of males was higher than that of females, irrespective of districts. When comparing geographical regions, the average hair Se content of southern residents was greater than that of northern residents, regardless of gender. Historically, the overall hair Se content of today’s inhabitants decreased between 24% and 46% when compared with the inhabitants living in the same geographic region 20 years ago. The decrease of hair Se content may be related to the overall decrease of grain consumption and the lower Se content in the staple food rice.

## 1. Introduction

Selenium is an essential trace element in human nutrition [1]. China, for example, is one of the 40 countries designated as low Se or Se deficient according to World Health Organization (WHO) [2]. The Se deficient areas account for 72% of the country’s total area, its deficiency affects over 70 million people who face the potential adverse health impacts due to Se deficiency [3]. Overt Se deficiency has caused serious health consequences in low Se areas of China, such as endemic Keshan disease (endemic cardiomyopathy) and Kaschin-Beck disease (endemic osteoarthropathy) [4]. Meanwhile, there is mounting evidence that suggests the importance of Se in the functioning of the immune system, counteracting the development of virulence, inhabiting HIV progression to AIDS, protecting against cardiovascular disease [1], mitigating and preventing the teratogenic effects exerted by such metals as Cd, Hg, Pb, and As [5], and even preventing the development of tumors and reducing the risk of some types of cancer [6].

Selenium enters the food chain through plants and the amount of Se in foods is directly affected by Se levels in the soil in which they are grown [5]. Intake of Se varies considerably between countries and regions of countries largely due to the variability of the Se content of plant foods from one part of the world to another. Grain, as staple food, plays a crucial role in the food supply in China, and it is also an efficient way to provide Se to consumers, *i.e.*, Korea [7], Japan [8]. In the last century, 70% of the Se intake of rural Chinese residents came from their staple diet [9]; after 2000, cereals were still major Se source food in the daily diet with the development of the economy, such as 23% in the Suzhou area [3], a developed area in China.

The concentration of Se in hair is a commonly-used index to evaluate body Se load, as it reflects the long-term Se level of the human body [10]. Hair Se had highly significant correlations with the Se concentration in muscle (*R* = 0.89, *n* = 15), whole blood (*R* = 0.90), red blood cells (*R* = 0.91), blood plasma (*R* = 0.87) and toenails (*R* = 0.85) [3,11]. Hair Se can also be used as an important indicator on endemic diseases and may play a critical role in the etiologic research on the Keshan disease, Kashin–Beck disease, and on local selenosis in China [12]. Furthermore, hair Se content was found to regularly increase from the Keshan disease zone (<0.20 μg/g) to the transition zone (0.20–0.25 μg/g) and to the non-disease zone (>0.25 μg/g) [13]. These values reflect the close relationship between the geographical distribution of hair Se and Keshan disease.

The present study aims: (1) to evaluate the Se nutritional status of Chinese residents by conducting a systematic survey on hair Se concentrations of residents crossing 10 provinces from northeast to southeast China; (2) to compare the Se nutrition of current residents with data reported in the past, and to analyze if the changes in hair Se content is due to reduced Se intake via rice as a staple food over time.

## 2. Materials and Methods

### 2.1. Sample Collection and Preparation

**Figure 1:**
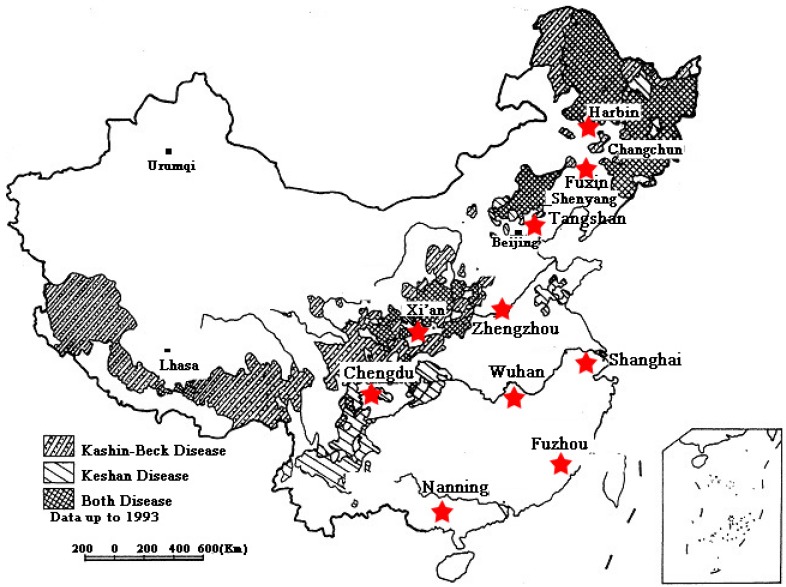
Distribution of KSD, KBD (by disease-affected countries) [4] in China, including the study areas within our survey (designated with red stars).

Hair and food samples were collected from the local residents in suburbs or cities. According to Se content in soil [4] we selected 10 provinces and municipalities extending from northeast to southeast China (Figure 1), including Se deficiency areas (Harbin in Heilongjiang province; Fuxin in Niaoling province; Tangshan in Hebei province; Zhengzhou in Henan province; Xian in Shaanxi province), and moderate Se areas (Shanghai municipality; Wuhan in Hubei province; Chengdu in Sichuan province; Fuzhou in Fujian province; and Nanning in Guangxi province). The selected areas are densely populated and have strong agriculture-driven economies. In every province, we selected three different residential locations to sample from urban and suburban populations, respectively, because it was impossible to practically sample on a large scale. In sampling, we balanced the sex ratio as best as possible, as well as age ratio: children:adults:seniors = 1:8:1. To avoid obvious bias, the detailed information was collected and taken into consideration for all residents before their ethics approval for sampling (*i.e.*, age, health, income, dwelling time). The study has been reviewed by the institutional review board of the Institute of Soil Science, Chinese Academy of Sciences and approved to proceed. Food samples were purchased from local supermarkets, including: rice, major types of vegetables, meats, and fishes. A total of 408 hair samples were collected from local healthy people of both genders ranging from 4 to 76 years old across the 10 provinces—there were 46 children (4–18 years old), 319 adults (19–60 years old) and 43 seniors (61–76 years old). Approximately 2.0 g of hair samples were cut between 1 and 3 cm from the nape of the neck. Hair samples were washed with acetone and distilled water three times, respectively, air-dried, stored, and cut into small pieces with stainless-steel scissors before digestion.

### 2.2. Sample Digestion

Samples of 0.5 g were weighed and placed into 50 mL PTFE digestion containers. Two mL of H_2_O_2_ were added and the sample container was shaken a few times to disaggregate the sample. Then, 6 mL of HNO_3_ were added and the PTFE container was shaken a few times again and mixed thoroughly. After sealing, the closed PTFE containers were left in the digestion vessel and placed at 160 °C for 8 h. The sample solution was then evaporated at 130 °C until a white fume formed, and the volume of the solution was reduced to 2 mL on the electric evaporation block. Acid fumes were removed by a water vacuum pump and adsorbed in a strong alkaline solution. After the solution was cooled to room temperature, 5 mL of 50% HCl were added to reduce selenate to selenite at 110 °C in the evaporation block for approximately two hours. As the solution became clear, it was transferred into a clean 15 mL polycarbonate tube, and filled to final volume with ultra-pure water. All hair samples were digested at least in duplicate. Two procedural (reagent) blanks were prepared with every sample digestion run. In addition, to minimize any possible contamination during sample digestion and AFS analysis, PTFE digestion vessels were washed with laboratory detergent, rinsed and soaked in 20% v/v HCl overnight, followed with a rinsing with ultra-pure water prior to sample digestion. Plastic containers, pipette tips, and test tubes used in the experimental work were soaked in 20% v/v HCl overnight, and lastly rinsed with ultra-pure water before use.

Ultra pure water (≥18.2 MΩ) from a Millipore Milli-Q system (Milford, MA, USA) was used for the preparation of all solutions. External calibration standards were prepared by gravimetric serial dilution from 1000 mg/L Se stock standard solution in 10% HCl (NCS^®^ analytical instruments, Beijing, China). The standard solutions were prepared at the beginning of each run with ultra-pure water and HCl (50% v/v) to a final 10% acid concentration. 2% (w/v) KBH_4_ was prepared using 0.5% (w/v) NaOH solution, and used as a reducing agent. All the chemical reagents used were super pure guarantee grade.

### 2.3. Determination of Selenium Concentration by HG-AFS

The Se concentration in each sample was determined by Hydride Generation Atomic Fluorescence Spectrometry (HG-AFS) 610D2 (Beijing Rayleigh Analytical Instrument Co., Ltd., Beijing, China). Instrumental conditions were as follows: lamp current 75 mA, photomultiple tube negative high voltage 300 v, atomizer height 87 mm, carrier gas flow rate 500 mL/min, and a shielding gas flow rate 100 mL/min. Procedural blanks were analyzed every two hours to monitor for variations in blank levels, and were measured after rinse solutions to minimize any memory effects.

The recovery and accuracy of the Se measurements were determinate with standard reference Se materials of rice (GBW10045), meat (GBW08552), cabbage (GBW10014) and human hair (GBW07601 GSH-1) prepared by the National Research Center for Standards in China, which contained 0.053 ± 0.014 mg Se/kg, 0.49 ± 0.05 mg Se/kg, 0.2 ± 0.03 mg Se/kg and 0.60 ± 0.04 mg Se/kg, respectively. The Se recovery was between 95% and 103%.

### 2.4. Statistical Analysis

Data were subjected to analysis of variance (ANOVA), and *post hoc* comparisons were performed with Duncan’s multiple range test at *p* < 0.05. The statistical software program used was SPSS version 13.0 (IBM, Armonk, NY, USA).

## 3. Results and Discussion

### 3.1. Hair Se Level of the Current Survey

**Figure 2:**
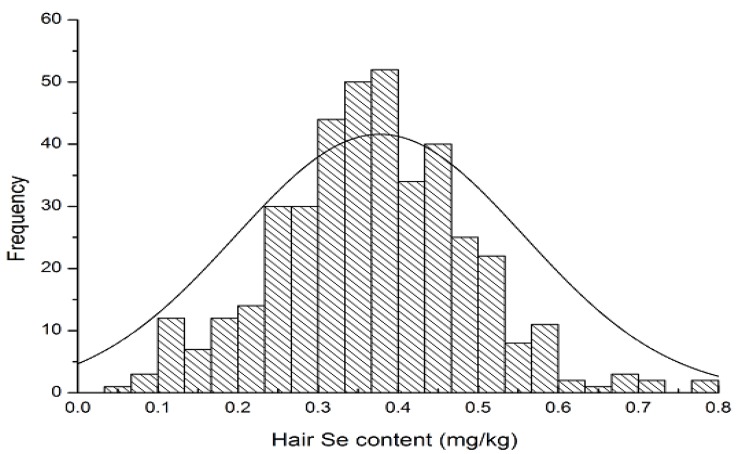
The statistical distribution of all residents’ hair Se concentrations in this survey.

The hair Se concentrations of 408 residents across 10 provinces and cities were analyzed statistically and they formed a normal distribution (Figure 2), in which most hair Se content ranged from 0.30 to 0.45 mg/kg, with a median value of 0.37 mg/kg. This concentration fell between normal hair Se values (0.36–0.74 mg/kg) as reported by Schroeder [14]. Moreover, the hair Se content of residents in this study was similar to results presented in recent reports of the same region in China, *i.e.*, 0.29 ± 0.08 in Heilongjiang mg/kg (Table 1) and 0.26 ± 0.08 mg/kg reported in 2011 [15], as well as 0.38 ± 0.16 mg/kg in Henan (Table 1) and 0.39 ± 0.10 mg/kg reported in 2008 for the Huaibei region [16] (one of the cities in the Anhui province adjacent to Henan). However, there was large individual variability in hair Se content of Chinese residents with a maximum value of 2.65 mg/kg and a minimum value of 0.06 mg/kg; a 46 times difference between maximum and minimum. There also existed a regional difference in the hair Se content among residents (Table 1); Shaanxi had the lowest median of 0.28 mg/kg, whereas Hubei had the greatest median of 0.44 mg/kg. In general, hair Se content of the northern residents was lower than that of the southern residents. The hair Se content in the northern areas (Heilongjiang, Liaoning, Hebei, Henan, and Shaanxi) was 0.29, 0.36, 0.38, 0.38, and 0.29 mg/kg, respectively, whereas hair Se content in the southern areas (Shanghai, Hubei, Sichuan, Fujian, and Guangxi) was 0.42, 0.44, 0.43, 0.40, and 0.39 mg/kg, respectively (Table 1).

Among the 408 survey participants, generally 342 (84%) residents had normal hair Se content according to hair Se ecological landscape value designated by past research efforts related to Se-endemic diseases in China [13]. The hair Se content of 35 residents (9%) was lower than 0.20 mg/kg, which was deemed to be in the Se deficiency status. The hair Se content of 31 residents (7%) ranged from 0.20 to 0.25 mg/kg and was considered to be on the edge of Se deficiency (Table 2). Two residents had hair Se content greater than 1.00 mg/kg, which was still below the value used to designate Se excess (3.00 mg/kg).

**Table 1:** Hair Se content of residents in different Chinese districts (mg/kg).

Districts	All residents				Males				Females			
	No.	Mean ± SD	Range	Median	No.	Mean ± SD	Range	Median	No.	Mean ± SD	Range	Median
Heilongjiang	30 ^‡^	0.29 ± 0.08 ^†^	0.14–0.50	0.29	15	0.33 ± 0.08	0.24–0.50	0.33	15	0.28 ± 0.08	0.14–0.40	0.27
Liaoning	50	0.36 ± 0.04	0.25–0.44	0.36	28	0.36 ± 0.04	0.25–0.44	0.36	22	0.31 ± 0.05	0.27–0.34	0.31
Hebei	37	0.38 ± 0.13	0.11–0.68	0.4	20	0.45 ± 0.10	0.24–0.68	0.45	17	0.29 ± 0.10	0.11–0.47	0.32
Henan	45	0.38 ± 0.16	0.10–0.96	0.37	20	0.44 ± 0.16	0.10–0.96	0.41	25	0.33 ± 0.15	0.08–0.57	0.32
Shaanxi	48	0.29 ± 0.12	0.10–0.78	0.28	31	0.30 ± 0.06	0.21–0.49	0.29	17	0.27 ± 0.16	0.10–0.78	0.26
Shanghai	47	0.42 ± 0.09	0.17–0.65	0.42	27	0.44 ± 0.08	0.24–0.55	0.45	20	0.39 ± 0.10	0.17–0.65	0.36
Hubei	36	0.44 ± 0.16	0.06–0.79	0.44	25	0.48 ± 0.10	0.29–0.72	0.47	11	0.33 ± 0.23	0.06–0.79	0.24
Sichuan	34	0.43 ± 0.24	0.22–0.60	0.4	15	0.52 ± 0.33	0.33-1.71	0.43	19	0.35 ± 0.09	0.22–0.56	0.32
Fujian	42	0.40 ± 0.37	0.16–0.52	0.37	28	0.58 ± 0.59	0.30–2.65	0.42	14	0.32 ± 0.11	0.08–0.52	0.31
Guangxi	39	0.39 ± 0.15	0.11–0.68	0.38	25	0.43 ± 0.10	0.18–0.54	0.39	14	0.32 ± 0.18	0.11–0.68	0.34
Total	408	0.38 ± 0.18	0.06–2.65	0.37	234	0.42 ± 0.20	0.10–2.65	0.39	174	0.32 ± 0.13	0.06–0.79	0.31

^‡^ No. is number of samples; ^†^ SD is standard deviation.

**Table 2:** Human hair Se content from 1985 to 2011 (mg/kg) in residents from different Chinese districts.

District	Province/City	Year	Sample Size	Average value	Range
Northern	Heilongjiang [17]	1995	1232	0.47 ± 0.20	
	Heilongjiang [15]	2011	27	0.26 ± 0.08	0.13–0.44
	Heilongjiang ^†^	2011	30	0.29 ± 0.08	0.14–0.50
	Liaoning [18]	1989	51	0.33 ± 0.03	
	Liaoning ^†^	2011	50	0.36 ± 0.04	0.25–0.44
	Jilin [19]	1993	10	0.86	0.79–0.93
	Beijing urban [20]	1985	311	0.57 ± 0.13	0.33–0.82
	Hebei ^†^	2011	37	0.38 ± 0.13	0.11–0.68
	Qinghai [21]	2006	2272	0.28 ± 0.15	0.04–0.731
	Inner Mongolia [22]	1995	400	0.76	0.04–2.24
	Shaanxi ^†^	2011	48	0.29 ± 0.12	0.10–0.78
	Henan [23]	1997	53	0.68 ± 0.20	
	Henan ^†^	2011	45	0.38 ± 0	0.08–0.96
Southern	Anhui [16]	2003	266	0.39 ± 0.10	
	Jiangsu [24]	1994	30	0.56 ± 0.16	
	Jiangsu [25]	2006	53	0.34	0.10–0.62
	Jiangsu [3]	2011	285	0.32, 0.39	
	Shanghai urban [26]	1986	200	0.72	0.29–1.8
	Shanghai urban [27]	1998	30	0.55	
	Shanghai urban^ †^	2011	24	0.38 ± 0.09	0.17–0.55
	Shanghai suburb [26]	1986	119	0.63	0.30–2.8
	Shanghai suburb ^†^	2011	23	0.45 ± 0.08	0.31–0.65
	Hubei [28]	1988	20	0.62 ± 0.08	
	Hubei ^†^	2011	18	0.47 ± 0.17	0.06–0.72
	Sichuan [29]	1992	107	0.51 ± 0.10	0.26–0.71
	Sichuan ^†^	2011	34	0.43 ± 0.24	0.22–1.71
	Guangdong [30]	2000	72	0.50 ± 0.12	
	Guangxi ^†^	2011	57	0.39 ± 0.14	0.11–0.79
	Fujian ^†^	2011	42	0.40 ± 0.37	0.08–2.65

^†^ Data from this survey.

### 3.2. Hair Se Differences between Males and Females

**Figure 3:**
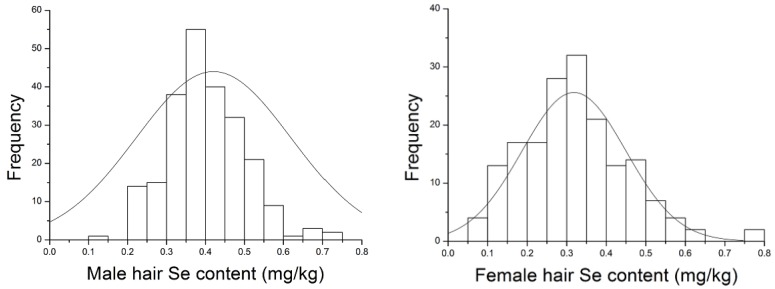
The statistical distribution of hair Se content in males and females.

This survey encompassed 234 males and 174 females. The hair Se concentration of both males and females represented a normal statistical distribution (Figure 3). Generally, the hair Se content of males was higher than that of females (Table 1). There existed significant difference (*p* < 0.05) between hair Se concentration of males and females, except for Liaoning and Shaanxi in the northern areas. Moreover, the hair Se content of males exhibited a more dense statistical distribution than that of females. Among the 234 males, only one had hair Se content lower than 0.20 mg/kg, and 14 (6%) males had hair Se content lower than 0.25 mg/kg. Among the 174 females, 34 (15%) had hair Se content lower than 0.20 mg/kg, while 17 (7%) had Se content less than 0.25 mg/kg. Thirty-eight males (16%) and 15 females (6%) had hair Se content greater than 0.50 mg/kg. The tendency of females to be Se deficient may be related to Se intake because females generally eat less, especially staple food, than males.

### 3.3. Hair Se Differences in Chinese Residents between Current Data and Historical Data

The hair Se content of residents in this survey was more similar to the results of studies conducted after 2000, but was significantly lower than that reported in the 1980s. In general, the hair Se content of residents reported before 2000 was greater than 0.50 mg/kg, whereas that reported after 2000 was lower than 0.50 mg/kg (Table 2). When comparing current survey data with past reported data in the same province, there were significant decreases. The hair Se level of Heilongjiang residents (0.29 mg/kg) was reduced by 37% from 0.47 mg/kg reported in 1995. The hair Se content of Henan residents (0.38 mg/kg) declined by 44% from 0.68 mg/kg reported in 1997. The hair Se content of Hubei residents (0.44 mg/kg) decreased by 30% from 0.62 mg/kg reported in 1987. The hair Se level of Sichuan residents (0.43 mg/kg) was 16% lower compared to 0.51 mg/kg reported in 1992. In Shanghai, the survey showed that the Se concentration of 0.39 mg/kg was 46% lower compared to 0.72 mg/kg reported in 1986, and 34% lower compared to 0.55 mg/kg reported in 1998. The hair Se level in the suburban areas of Shanghai (0.45 mg/kg) decreased by 28% from 0.63 mg/kg reported in 1985, which represents a smaller degree of change compared with that in urban areas. In a similar long-term survey, Lyons also found a decline in human blood and plasma Se status in South Australia from the late 1970s to 2003 [31].

### 3.4. Factors that Influence Variations of Hair Se Content

**Figure 4:**
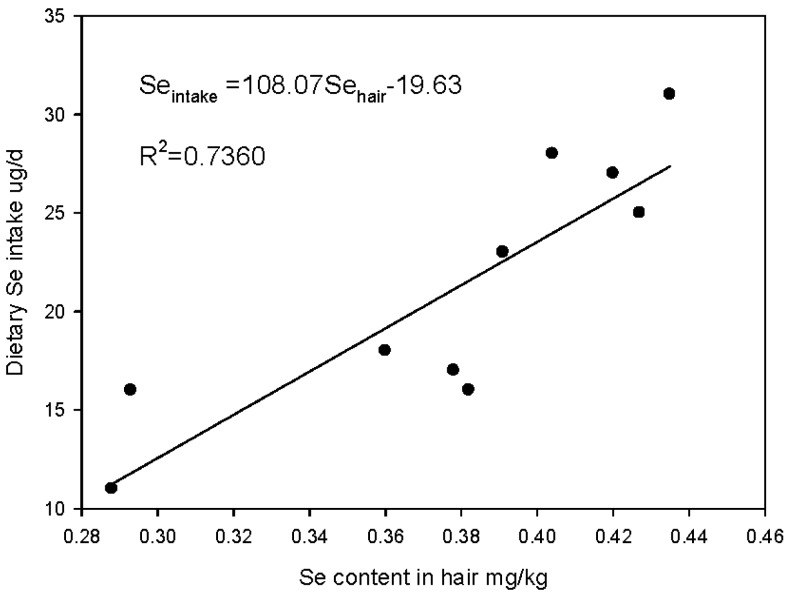
The relationship between daily Se intake and hair Se content in different districts (*n* = 10).

The concentrations of Se in rice, vegetables, meats, and fishes collected from the tested provinces above were determined as described in Table 3. According to the dietary structure and Se concentration of different foods of every province (reported from Chinese statistical yearbook 2011), the estimated daily Se intake per capita of every province varied from 11 to 31 μg day^−1^ (see Table 3). Moreover, there was significant linear correlation (*R* = 0.86, *n* = 10) between daily Se intake of every province and corresponding hair Se content (Figure 4). The Se supplied by grains greatly influences the Se intake of residents. Assuming rice as the main source of grain, daily Se intake from rice was 34%–57% as the major food source, which significantly contributed to the daily human Se intake. In this survey, Se concentration of rice ranged from 19 to 31 μg/kg, and the national average value of 24 μg/kg was less than the data reported in both of the other regions [3] and slightly less than the average Se concentration of 25 μg/kg in regular rice [32]. Furthermore, the Chinese residents dramatically reduced their rice consumption from 1985 to 2011 (Chinese statistical yearbook from 1985 to 2011). Using the rice average Se concentration to estimate the change of Se intake, the average Se intake from rice went from 9 μg/day in 1985 to 6 μg/day in 2011, indicating a decline of 40% (Table 4). According to the acceptable least daily intake of 50 μg Se recommended by the Chinese Nutrition Society for adults, the Se intake supplied by rice accounted for 18% of the least daily intake in 1985, and only 11% in 2011. Calculations show that the daily Se intake of adults only ranges from 26 to 32 μg in China [33], and the decrease of Se intake from grains constitutes a much larger proportion of the total dietary Se intake of adults. Similarly, the decrease of staple Se content contributed to a decrease of Se intake in the UK, where the Se intake fell from 60 to 63 ug/day to the current level of 34–39 ug/day [34]. This lower intake may be due to the reduced importation of North American (largely Canadian) wheat for bread-making, which was higher in Se content than the lower Se-containing EU wheat varieties.

**Table 3:** Selenium content in different foods and calculated daily Se intake for different Chinese districts.

Districts	Se Concentration in Foods (μg/kg)				Food Consumption (g) ^‡^				Se Intake (μg/day)
	Rice ^†^	Vegetable	Meat	Fish	Grain	Vegetable	Meat	Fish	
Heilongjiang	21	4.2	93	266	416	225	29	12	16
Niaoning	20	4.5	86	249	472	306	41	14	18
Hebei	23	7.7	91	263	456	216	26	9	17
Henan	22	7.6	106	283	393	188	18	5	16
Shaanxi	20	4.8	78	269	418	144	21	2	11
Shanghai	19	5.5	111	234	396	190	59	51	27
Hubei	30	6.1	152	348	445	361	49	23	31
Sichuan	31	6.7	103	209	456	313	73	7	25
Fujian	31	9.9	97	252	463	236	20	46	28
Guangxi	25	9.8	109	276	490	240	46	11	23

**^†^** Se concentration expressed on a dry weight basis; **^‡^** food consumption originated from Chinese statistical yearbook 2011.

**Table 4:** The Se concentration of rice and Se intake from grain.

	The Se Intake from Grain of Urban Residents (μg/day) ^†^						
Year	1985	1990	1995	2000	2005	2010	2011
Grain consumption (g/person/day)	369	358	266	226	211	223	221
Se intake from grain (μg/day, in the national average)	9	9	7	6	5	6	6
Percent of lowest daily intake for adult (%) ^‡^	18	18	13	11	11	11	11

**^†^** Food consumption of urban residents originated from Chinese statistical yearbook 1985–2011; ^‡^ According to the lowest daily Se intake, 50 μg is recommended by Chinese Nutrition Society for adults.

## 4. Conclusions

The survey results of the hair Se content presently show the variability among individuals, gender, and regional differences in residents from northeast to southeast China. The hair Se content of today’s residents decreased by 24%–46% compared with past residents in the same geographic region. The decrease of hair Se content may be primarily related to the decrease of grain consumption and the lower Se content in the staple food rice.
